# Enhanced Low-Energy Gamma-Ray Shielding Performance of Nano ZrO_2_-Reinforced Vinyl Ester Composites: MCNP6.3, XCOM, and Phy-X/PSD Assessment

**DOI:** 10.3390/ma19214489

**Published:** 2026-10-25

**Authors:** Seda Erdönmez

**Affiliations:** Department of Civil Engineering, Faculty of Engineering and Architecture, Istanbul Gelisim University, Istanbul 34310, Turkey; serdonmez@gelisim.edu.tr

**Keywords:** gamma-ray shielding, vinyl ester, zirconia nanoparticles, MCNP6.3, XCOM, Phy-X/PSD, mass attenuation coefficient, effective atomic number, buildup factors

## Abstract

**Highlights:**

- ZrO_2_ addition improved attenuation most strongly at low photon energies.
- At 81 keV, VE–Zr20 raised MAC by 104.1% and lowered HVL by 58.1%.
- MCNP6.3 agreed with XCOM and Phy-X/PSD, with a 0.78% mean relative deviation.
- Peak EBF and EABF decreased with ZrO_2_ loading; peak EBF fell by 51% at 0.5 MFP.
- VE–Zr20 provided the best low-energy shielding among the tested compositions.
- VE–Zr20 had only 11.9% of lead’s density, supporting lightweight shielding designs.
- The lead-free formulation may reduce health and environmental concerns linked to lead shields.

**Abstract:**

Vinyl ester (VE) nanocomposites containing 0–20 wt.% ZrO_2_ relative to the resin were produced to assess the effect of ZrO_2_ loading on gamma-ray shielding. The crystalline structure and morphology of the ZrO_2_ nanoparticles were examined by X-ray diffraction and transmission electron microscopy, respectively. Photon transport was simulated using MCNP6.3 at five energies associated with ^133^Ba, ^137^Cs, and ^60^Co, with results benchmarked against XCOM and Phy-X/PSD. The MCNP6.3-derived mass attenuation coefficients (MACs) agreed closely with both methods, with a mean relative deviation of 0.78% in each comparison. ZrO_2_ incorporation generally increased the effective atomic number (*Z*_eff_) and reduced the half-value layer (HVL) and mean free path (MFP), with the strongest effects at low energies. At 81 keV, VE-Zr20 exhibited a 104.1% higher MAC and a 58.1% lower HVL than VE-Zr0, while differences among the composites diminished at higher energies. Peak exposure buildup factor (EBF) and energy absorption buildup factor (EABF) values increased with depth but decreased with ZrO_2_ content; at 0.5 MFP, the maximum EBF decreased by 51%, from 3.18 (VE-Zr0) to 1.55 (VE-Zr20). VE-Zr20 provided the most favorable low-energy shielding performance, supporting the potential use of ZrO_2_-reinforced VE composites as lightweight, lead-free shielding materials for applications involving low-energy photons.

## 1. Introduction

Ionizing radiation is widely used in food processing, diagnostic imaging, radiotherapy, and nuclear power generation [1,2]. However, excessive exposure can cause serious health effects, including cancer, DNA damage, and infertility [3]. Effective radiation protection is therefore essential in these fields. Shielding materials are commonly used to reduce the radiation dose received by individuals, and their development and performance have been extensively investigated in the literature [4,5,6,7,8,9,10,11,12,13,14,15,16,17,18,19]. However, conventional shielding materials such as lead and concrete have several limitations, including high density, limited flexibility, lead toxicity, and the susceptibility of concrete to cracking [20]. Polymer-based composites have therefore gained considerable attention as lightweight, readily processable, and compositionally adaptable alternatives to conventional lead-based materials. Their radiation attenuation performance can be improved by incorporating suitable inorganic fillers, including compounds containing high-atomic-number (high-*Z*) elements. The extent of this improvement depends on photon energy, the elemental composition and concentration of the filler, and the density of the composite. Accordingly, the photon shielding performance of numerous polymer–filler systems with different filler types and concentrations has been evaluated experimentally and theoretically [21,22,23,24].

Within the broader class of polymer-based shielding materials, vinyl ester (VE) is an attractive thermosetting matrix because it combines the processability of unsaturated polyester resins with the mechanical strength, adhesion, and chemical resistance associated with epoxy resins [25]. Nevertheless, studies focusing specifically on VE-based radiation shielding composites remain relatively limited. El-Toony et al. prepared VE nanocomposites containing 50 wt.% Pb_2_O_3_ and reported that, at 662 keV and a thickness of 4 cm, the attenuation percentage reached 86%, compared with 32% for neat VE [26]. However, the use of a lead-containing filler raises concerns regarding toxicity and potential environmental release. Akkurt et al. evaluated VE–pumice composites containing up to 50 wt.% pumice over 511–2140 keV using MCNPX and FLUKA, with the composite containing 50 wt.% pumice (V–P50) showing the most favorable shielding performance [27]. Günoğlu et al. evaluated VE composites containing 0.5–2 wt.% boron nitride (BN) at 662–1332 keV through experimental measurements and XCOM calculations, reporting an approximately 41% increase in the linear attenuation coefficient (LAC) at 1332 keV relative to neat VE [28]. In a subsequent experimental and theoretical study, the LAC of colemanite-filled VE composites similarly increased with filler content [25]. More recently, Erdönmez evaluated VE composites containing 2.5–20 wt.% CeO_2_ nanoparticles using experimental measurements, MCNP6 simulations, and WinXCOM calculations, reporting that the mass attenuation coefficient (MAC) of the 20 wt.% CeO_2_ composite at 81 keV was approximately 5.3 times that of neat VE [29].

ZrO_2_ is a robust ceramic material that combines high thermal stability and mechanical strength with strong resistance to wear, corrosion, and chemical degradation [30,31,32]. Previous studies have highlighted the potential of ZrO_2_ as a functional filler in lead-free shielding systems based on both glass and polymer matrices. For example, Abouhaswa et al. incorporated 0–4 mol% ZrO_2_ into lithium bismuth borate glasses by melt quenching and reported a MAC of 1.564 cm^2^/g at 81 keV for the glass containing 4 mol% ZrO_2_ [33]. Abdelghany et al. evaluated borotellurite glasses containing 0–3 mol% ZrO_2_ using XCOM and Phy-X/PSD and reported a MAC of 0.1326 cm^2^/g at 280 keV for the glass containing 3 mol% ZrO_2_ [34]. Elsafi et al. prepared epoxy composites containing 10–40 wt.% ZrO_2_ and 15 wt.% B_2_O_3_ and reported an LAC of 2.978 cm^−1^ at 60 keV for the composite containing 40 wt.% ZrO_2_ [35]. However, existing ZrO_2_-based shielding studies have mainly focused on glass and epoxy matrices, whereas the energy-dependent shielding characteristics of VE–ZrO_2_ composites have not yet been systematically investigated.

Taken together, the mechanical strength, chemical resistance, and processability of VE and the thermal stability and demonstrated photon attenuation capability of ZrO_2_ make VE–ZrO_2_ composites promising candidates for lead-free radiation shielding applications. Accordingly, VE–ZrO_2_ composites containing 0–20 wt.% ZrO_2_ were produced, and their gamma-ray attenuation characteristics were systematically investigated for the first time. The MAC values obtained from Monte Carlo N-Particle Transport (MCNP6.3) simulations at selected photon energies were compared with the corresponding values calculated using XCOM and Phy-X/PSD. The effective atomic number (*Z*_eff_), half-value layer (HVL), and mean free path (MFP) were also evaluated, together with the exposure buildup factor (EBF) and energy absorption buildup factor (EABF) at different penetration depths. The findings may support the further development of these composites as lightweight, lead-free materials for low-energy photon shielding applications, such as protective panels, equipment enclosures, and shielding liners for diagnostic X-ray units and laboratory setups involving low-energy photon sources.

## 2. Materials and Methods

### 2.1. Materials

For the production of the VE–ZrO_2_ composites, Polives 701, a bisphenol A epoxy-derived vinyl ester resin containing 35 wt.% styrene, was supplied by Poliya (Istanbul, Turkey), while ZrO_2_ nanoparticles (purity: 99.95%, average particle size: 30 nm) were obtained from Nanografi (Ankara, Turkey). For the curing of the vinyl ester matrix, methyl ethyl ketone peroxide (MEKP, C_8_H_18_O_6_) was used as the initiator, and cobalt octoate (6% Co, CoC_16_H_30_O_4_) was employed as the accelerator; both were purchased from Akpa Kimya (Istanbul, Turkey).

### 2.2. Characterization of ZrO_2_ Nanoparticles

**Figure 1:**
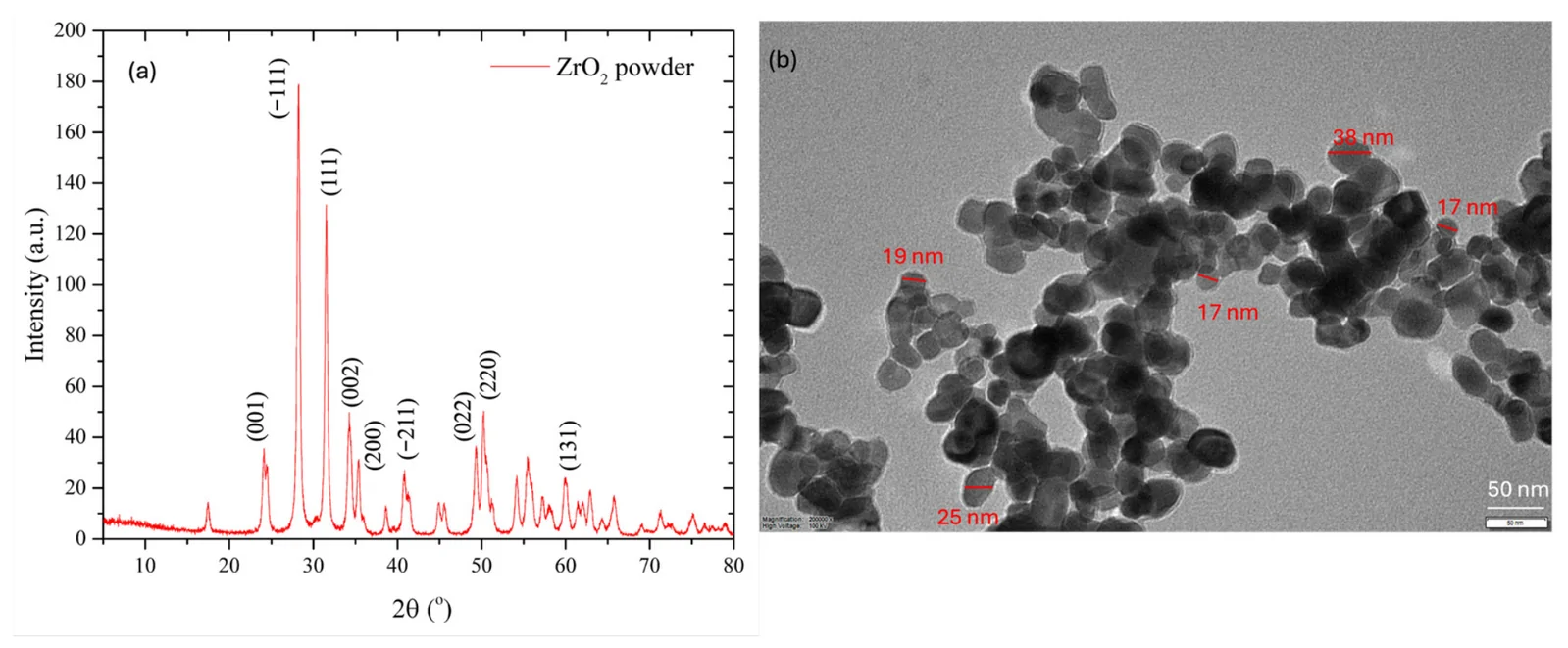
XRD spectrum (**a**) and TEM image (**b**) of the ZrO_2_ nanoparticles.

The crystal structure of ZrO_2_ nanoparticles was examined by X-ray diffraction with a Bruker X-ray diffractometer equipped with a LynxEye detector (Bruker AXS GmbH, Karlsruhe, Germany) and Cu Kα radiation (λ = 1.5406 Å). Data were collected in coupled 2θ/θ geometry over 5–80°, with a step size of approximately 0.02°, at 30 kV and 10 mA. Figure 1a presents the diffraction pattern recorded for the ZrO_2_ particles. Characteristic diffraction peaks observed at 2θ = 24.07°, 28.21°, 31.48°, 34.18°, 35.34°, 40.77°, 49.29°, 50.15°, and 59.81° were assigned to the (011), (−111), (111), (002), (200), (−211), (022), (220), and (131) crystallographic planes, respectively. The observed diffraction peaks matched well with JCPDS card No. 37-1484, which confirmed the presence of the monoclinic phase of ZrO_2_ [36,37]. The crystallite size was also estimated using the Debye–Scherrer formula given in Equation (1).

(1) $$D=\frac{k\lambda }{\beta cos\theta }$$

where *D* denotes the crystallite size, *k* is the Scherrer shape factor (0.89), and *λ* refers to the wavelength of the Cu Kα radiation. The parameter *β* represents the full width at half maximum (FWHM) of the selected peak expressed in radians, whereas *θ* is the associated Bragg angle. Using the most intense diffraction peak located at 2*θ* = 28.21°, assigned to the (−111) crystallographic plane, the crystallite size was estimated to be 18.75 nm.

ZrO_2_ nanoparticle morphology was examined by TEM (JEOL JEM-1220; 100 kV). As shown in Figure 1b, the ZrO_2_ particles exhibited generally rounded to quasi-spherical shapes, although some irregularly shaped particles were also observed. The particles appeared in closely packed clusters, suggesting a tendency toward agglomeration. Representative measurements of selected particles indicated apparent dimensions of approximately 17–38 nm, with the larger values possibly reflecting particle overlap. These dimensions were within the same nanometer range as the XRD-estimated crystallite size of 18.75 nm and were reasonably consistent with the nominal average particle size of 30 nm specified by the manufacturer.

### 2.3. Production and Physical Properties of VE–ZrO_2_ Nanocomposites

The direct pouring method was used to produce pure VE and VE–ZrO_2_ nanocomposites. The samples were designated as VE-ZrX, where X represents the ZrO_2_ loading (wt.%) relative to the VE resin; accordingly, VE-Zr0 denotes the pure VE sample.

**Figure 2:**
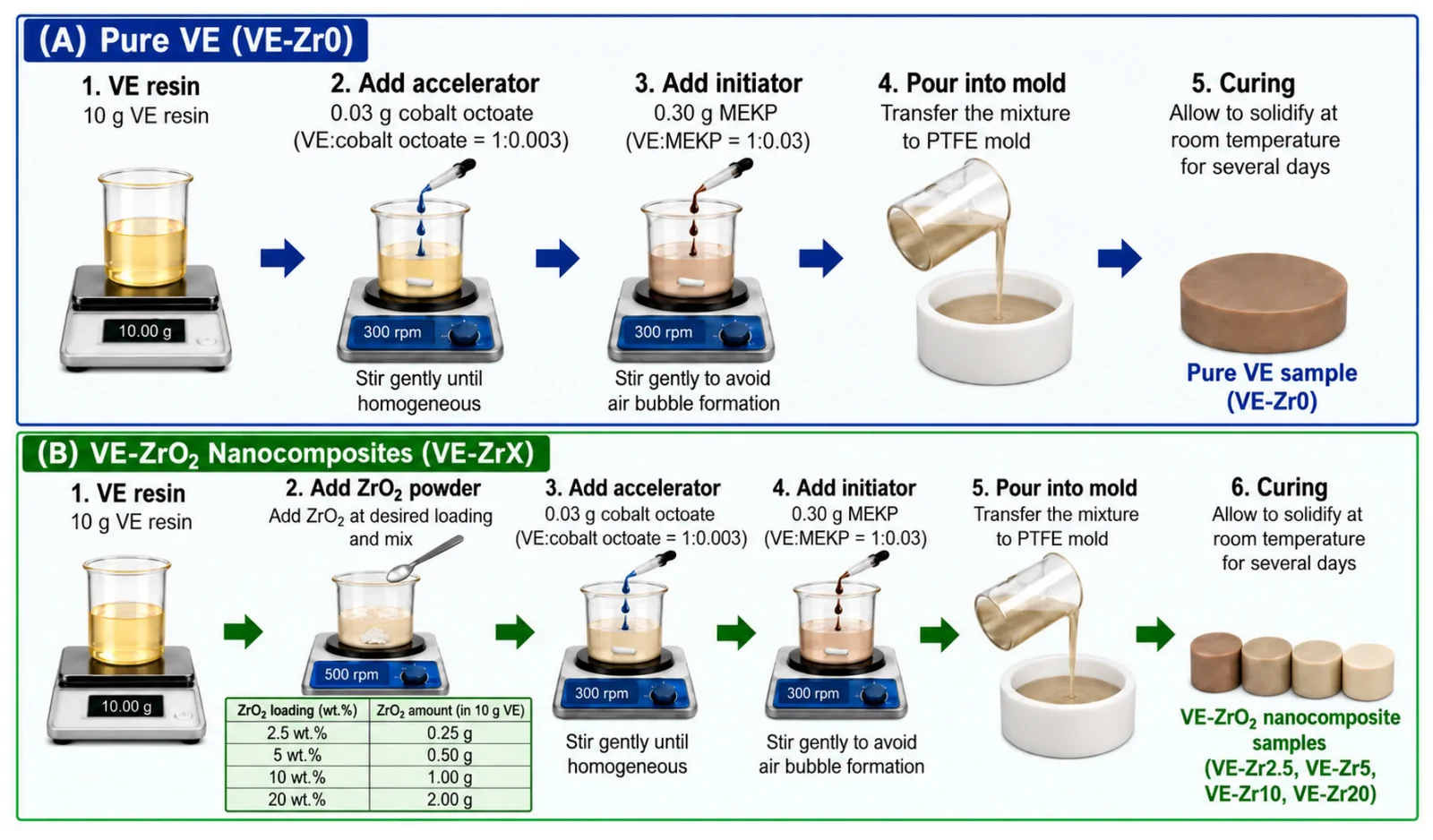
Schematic illustration of the direct-pouring production procedure for (**A**) pure vinyl ester (VE-Zr0) and (**B**) ZrO_2_-reinforced vinyl ester nanocomposites (VE-ZrX) with ZrO_2_ loadings of 2.5, 5, 10, and 20 wt.% relative to the VE resin.

In the first step of the synthesis procedure, 10 g of VE resin was placed in a beaker. The cobalt octoate (CoOct) accelerator was then added to the VE resin at a VE:CoOct weight ratio of 1:0.003, corresponding to 0.03 g of cobalt octoate, under gentle mechanical stirring until a homogeneous mixture was obtained. Subsequently, MEKP curing initiator was added to the resulting mixture at a VE:MEKP weight ratio of 1:0.03, equivalent to 0.30 g of MEKP, followed by slow stirring to minimize air entrapment. The prepared mixture was transferred to a PTFE mold and allowed to solidify at room temperature for several days, as schematically illustrated in Figure 2A.

For the preparation of VE–ZrO_2_ nanocomposites, ZrO_2_ powder was mixed with the vinyl ester resin. ZrO_2_ was incorporated into the composites at loadings of 2.5, 5, 10, and 20 wt.% relative to the VE resin, corresponding to 0.25, 0.50, 1.00, and 2.00 g of ZrO_2_ per 10 g of VE, respectively. Subsequently, the same procedure, including the incorporation of 0.03 g of cobalt octoate and 0.30 g of MEKP, was followed to prepare the nanocomposites, as illustrated in Figure 2B.

**Figure 3:**
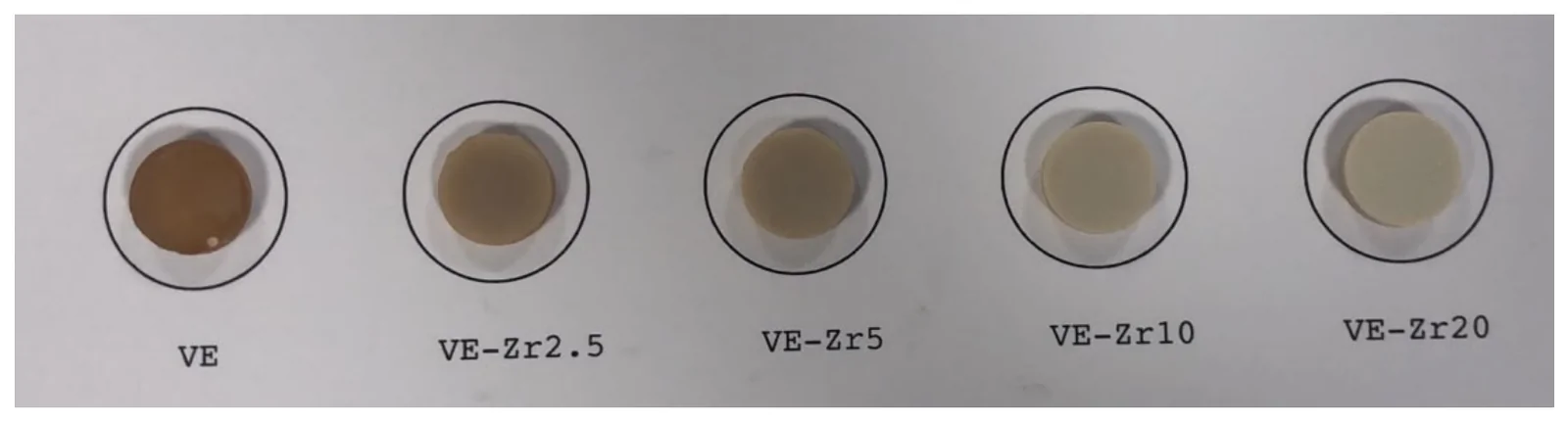
Photographs of the produced pure VE and VE–ZrO_2_ nanocomposites.

Photographs of the produced pure VE and VE-ZrO_2_ nanocomposites are also presented in Figure 3. All samples had a cylindrical (disk-shaped) geometry. Dimensional measurements showed that the diameters of the samples ranged from 17.61 to 17.91 mm and their thicknesses ranged from 3.63 to 4.74 mm. The densities of all specimens were experimentally measured according to Archimedes’ principle using a Weightlab WSA-224T analytical balance (Weightlab Instruments, Istanbul, Turkey) with a readability of 0.1 mg. For the measurements, each specimen was initially weighed in air, followed by weighing while submerged in distilled water. The density values were obtained directly from the instrument’s density measurement function, which is based on the relationship given in Equation (2).

(2) $$\rho =\frac{W_{air}}{W_{air}-W_{water}}\rho_{water}$$

where *W*_air_ represents the specimen weight in air, *W*_water_ denotes the apparent sample weight during immersion in distilled water, and *ρ*_water_ is the water density corresponding to the test temperature. As shown in Table 1, the experimentally measured density progressively increased from 1.152 g/cm^3^ to 1.349 g/cm^3^ with increasing ZrO_2_ nanoparticle content. This systematic increase is consistent with the incorporation of ZrO_2_, which has a substantially higher intrinsic density than the VE matrix, thereby increasing the overall density of the resulting nanocomposites. The measured densities agreed closely with theoretical densities calculated using the mixture rule [38], with deviations below 2% for all composites. The minor differences may be attributed to measurement uncertainty and the exclusion of the initiator and accelerator from the theoretical calculations. The experimentally determined densities were subsequently used to calculate density-dependent shielding parameters, including the LAC, HVL, and MFP, and were also specified as material inputs in the MCNP6.3 simulations. Table 1 also presents the elemental weight fractions, experimental and theoretical densities, and relative heaviness (RH) values of the investigated samples. RH expresses the experimental density of a composite as a percentage of the density of lead and was calculated using Equation (3).

(3) $$RH\left(\%\right)=\frac{\rho_{exp}}{\rho_{Pb}}\cdot 100$$

where *ρ*_exp_ is the experimental density of the composite and *ρ*_Pb_ is the density of lead (11.34 g cm^−3^).

Relative heaviness increased from 10.16% for VE-Zr0 to 11.90% for VE-Zr20 with increasing ZrO_2_ content, while all composites remained considerably less dense than lead. This substantially lower density may offer a practical advantage in shielding applications where reducing component weight is important. The elemental weight fractions were used to define the material compositions in MCNP6.3 and as inputs to XCOM and Phy-X/PSD.

### 2.4. MCNP6.3 Simulation

**Figure 4:**
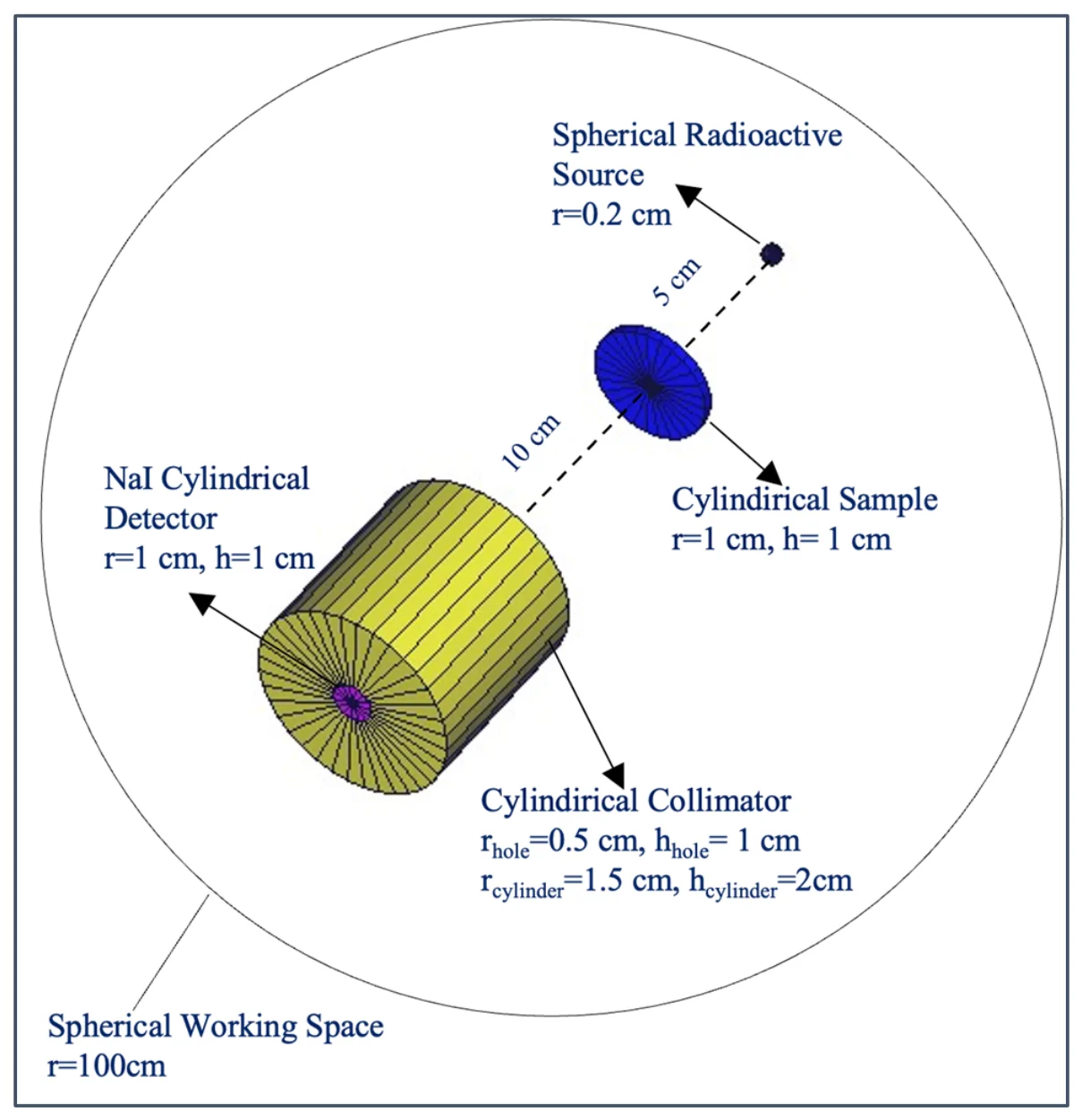
Geometry of the MCNP6.3 simulation model showing the spherical photon source, cylindrical specimen, lead collimator, and detector.

Gamma-ray attenuation in the investigated VE–ZrO_2_ nanocomposites was modeled through MCNP6.3 Monte Carlo simulations (Los Alamos National Laboratory, Los Alamos, NM, USA). Developed at Los Alamos National Laboratory, MCNP6.3 follows the transport of photons, neutrons, and electrons through material systems [39]. In this study, it was used to model photon interactions with the investigated composites. A schematic representation of the simulation setup, which includes the detector, photon source, and specimen, is given in Figure 4. A vacuum sphere extending 100 cm from its center defined the simulation region. Simulation geometry included a monoenergetic spherical photon source (r = 0.2 cm), along with a cylindrical specimen and detector, each defined by (r = h = 1) cm. Source–specimen and specimen–detector separations were 5 and 10 cm, respectively (Figure 4). To restrict off-axis photons and reduce scattered-photon contributions, a cylindrical lead collimator was placed around the detector. The cylindrical lead collimator had an outer radius of 1.5 cm and a total height of 2 cm. Its central opening had a radius of 0.5 cm and a height of 1 cm. The F4 tally provided the photon flux averaged over the detector cell. Photon transport calculations employed MCPLIB04 cross-section data derived from the ENDF/B-VI Release 8 evaluation. A reference simulation was performed to determine the unattenuated photon flux (I_0_) at the detector. Subsequently, simulations were carried out for the specimens with different elemental compositions listed in Table 1 at photon energies of 81, 356, 662, 1173, and 1332 keV. These energies correspond to gamma-ray emissions from ^133^Ba, ^137^Cs, and ^60^Co radioactive sources and span a broad photon-energy range. For each specimen, the LAC was derived from the ratio of the incident photon flux, I_0_, to the transmitted photon flux, I, according to the Beer–Lambert law. The coefficient was expressed as μ = ln(I_0_/I)/x, with x denoting the specimen thickness. The corresponding MAC was obtained as μ/ρ, where ρ is the specimen density. Each calculation tracked 10^7^ particle histories. The computational system ran 64-bit Windows 10 and used a 12-core Intel^®^ Core™ i7-12700H processor operating at 2.30 GHz together with 16 GB of memory. The relative statistical error of the F4 tally remained below 0.05% in all simulations.

### 2.5. Theoretical Calculation of Radiation Shielding Parameters

The attenuation behavior of pure VE (VE–Zr0) and the ZrO_2_-filled nanocomposites (VE–Zr2.5, VE–Zr5, VE–Zr10, and VE–Zr20) was also examined theoretically through two computational resources: XCOM, maintained by NIST, and the online Phy-X/PSD platform. XCOM contains photon-interaction cross sections and attenuation coefficients covering energies from 1 keV to 100 GeV for individual elements as well as compounds and mixtures [40]. Şakar et al. introduced Phy-X/PSD as a web-based tool for dosimetry and radiation-shielding calculations [41]. Using this platform, MAC, LAC, HVL, MFP, and *Z*_eff_ can be determined between 1 keV and 100 GeV, whereas EBF and EABF calculations cover 0.015–15 MeV. Brief descriptions of the parameters examined here are provided below.

MAC (cm^2^ g^−1^) quantifies photon attenuation per unit mass, whereas LAC (cm^−1^) describes attenuation per unit thickness and is related to MAC through Equation (4) [42].

(4) LAC=MAC·ρ

HVL corresponds to the thickness at which the transmitted photon intensity falls to one-half of its initial value. It was obtained from Equation (5) [43].

(5) $$HVL=\frac{\ln2}{LAC}$$

MFP represents the mean separation between two consecutive photon interactions within the shielding medium. It was obtained from Equation (6).

(6) $$MFP=\frac{1}{LAC}$$

*Z*_eff_ is an essential parameter for characterizing the photon shielding capability of a shielding sample. In the present study, *Z*_eff_ values were determined by interpolation using XCOM-derived MAC data over the full energy range and MCNP6.3-derived MAC data at the five selected photon energies. First, the effective atomic cross section (*σ*_eff_) was determined using Equation (7). Subsequently, *Z*_eff_ values were obtained by interpolating between the elemental atomic cross sections (*σ*_1_ and *σ*_2_) that bracket *σ*_eff_, using their corresponding atomic numbers (*Z*_1_ and *Z*_2_), according to Equation (8).

(7) $$\sigma_{eff}=\frac{{\left(\mu /\rho \right)}_{material}}{N_{A}\sum f_{i}/A_{i}}$$

In this expression, *N*_A_ denotes the Avogadro constant, *f_i_* corresponds to the weight fraction of the *i*th element, and *A_i_* refers to the atomic mass of that element [44].

(8) $$Z_{eff}=\frac{Z_{1}\left(log\sigma_{2}-log\sigma_{eff}\right)+Z_{2}(log\sigma_{eff}-log\sigma_{1})}{log\sigma_{2}-log\sigma_{1}}$$

Buildup factors describe the effect of scattered photons on the radiation response inside a shielding sample. EBF represents the buildup in photon exposure caused by multiple scattering, whereas EABF describes the buildup in energy absorption within the medium. In Phy-X/PSD, these factors are evaluated using the geometric progression (G-P) fitting method based on the ANSI/ANS-6.4.3 database [45]. Although the EBF and EABF values in the present study were obtained directly from Phy-X/PSD, the underlying theoretical procedure is summarized below.

The equivalent atomic number, *Z*_eq_, was established for each composite and photon energy from a quantity formed by dividing the Compton partial MAC by the corresponding total MAC:

(9) $$R=\frac{{\left(\mu /\rho \right)}_{compton}}{{\left(\mu /\rho \right)}_{total}}$$

For each energy, elemental *R* data were used to bracket the value calculated for the composite. *R*_1_ and *R*_2_ denote the two bounding values, while *Z*_1_ and *Z*_2_ identify the atomic numbers of the neighboring elements. *Z*_eq_ was then evaluated through logarithmic interpolation:

(10) $$Z_{eq}=\frac{Z_{1}\left(logR_{2}-logR\right)+Z_{2}(logR-logR_{1})}{logR_{2}-logR_{1}}$$

After establishing *Z*_eq_, the values of the G-P coefficients *a*, *b*, *c*, *d*, and *X_k_* for the composite were derived from those of the two neighboring elements on a logarithmic scale:

(11) $$P=\frac{P_{1}\left(logZ_{2}-logZ_{eq}\right)+P_{2}(logZ_{eq}-logZ_{1})}{logZ_{2}-logZ_{1}}$$

where *P* denotes any of the G-P fitting parameters *a*, *b*, *c*, *d*, or *X_k_*, while *P*_1_ and *P*_2_ are the corresponding coefficients for the elements with atomic numbers *Z*_1_ and *Z*_2_, respectively. The buildup factor *B*(*E*,*x*) at photon energy *E* and penetration depth *x*, expressed in MFP, is then calculated using Equations (12) and (13).

(12) $$B\left(E,x\right)=1+\frac{b-1}{K-1}\left(K^{x}-1\right)\;for\;K\neq 1$$

(13) $$B\left(E,x\right)=1+\left(b-1\right)x\;for\;K=1$$

where *b* represents the buildup factor at 1 mean free path (MFP), and *K*(*E*,*x*) is determined using Equation (14).

(14) $$K\left(E,x\right)=cx^{a}+d\frac{\tanh\left(\frac{x}{x_{k}}-2\right)-tanh(-2)}{1-tanh(-2)}x\leq 40\;MFP$$

In this work, the EBF and EABF values of the VE–ZrO_2_ composites were obtained from Phy-X/PSD for photon energies between 0.015 and 15 MeV at depths of 0.5, 1, 5, 10, 20, and 40 MFP. The resulting values were used to evaluate the multiple-scattering behavior and energy absorption characteristics of the investigated composites.

## 3. Results

**Figure 5:**
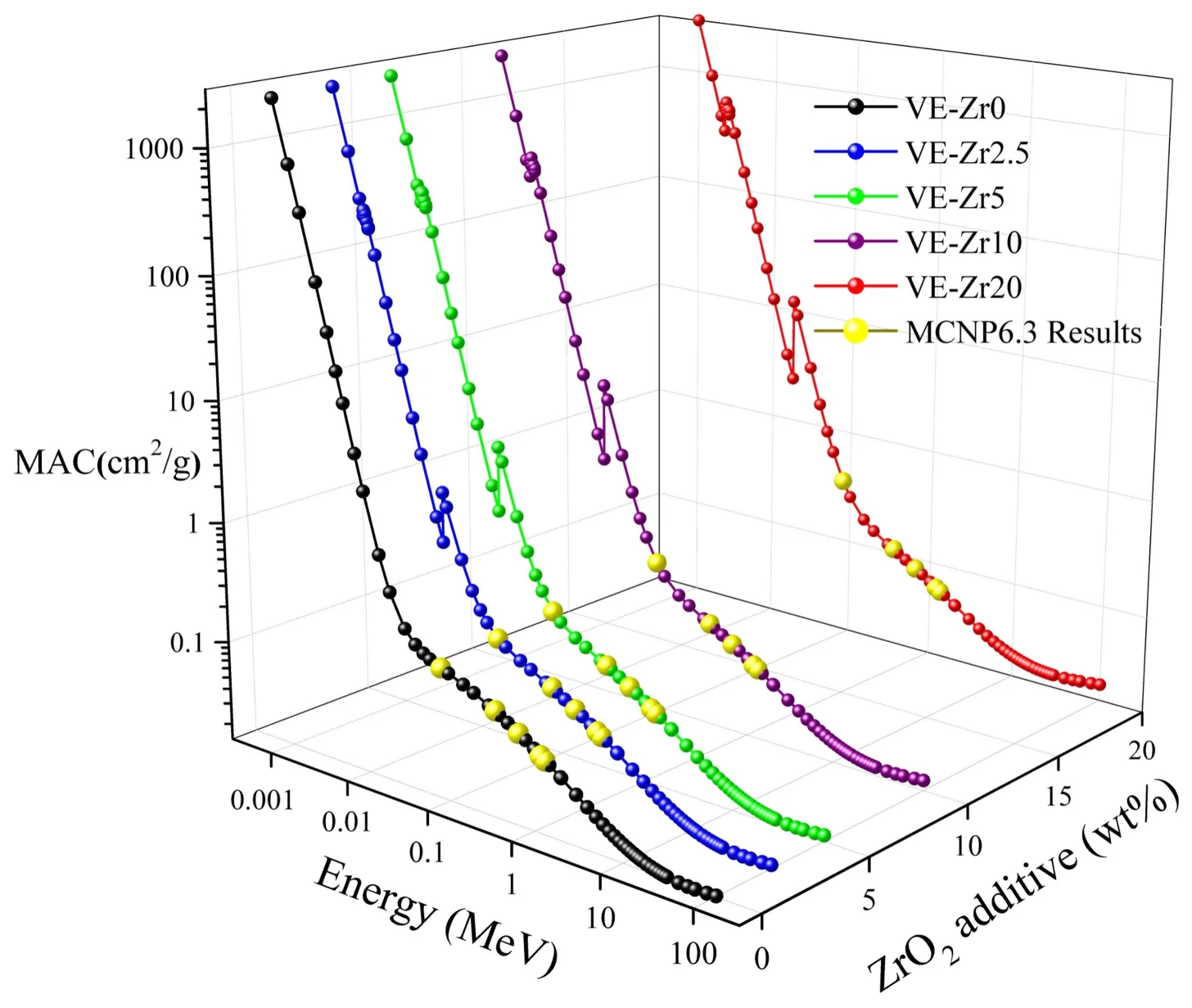
Energy-dependent MAC values of the VE–ZrO_2_ composites obtained from XCOM calculations (solid lines), together with the corresponding MCNP6.3 results at selected photon energies (yellow symbols).

The gamma-ray attenuation performance of the pure VE and the VE–ZrO_2_ nanocomposites was evaluated using the MCNP6.3 simulations, the XCOM database, and the Phy-X/PSD platform. The XCOM-derived MAC values spanning 0.001–100 MeV are displayed in three dimensions in Figure 5, alongside MCNP6.3 results at selected photon energies.

Figure 5 shows that the MAC values generally decrease with increasing photon energy. Increasing ZrO_2_ loading produces a pronounced enhancement primarily in the low-energy region, whereas the differences among the composites diminish at higher photon energies. Among the investigated compositions, VE-Zr20 exhibited the highest MAC values in the low-energy region, demonstrating the beneficial effect of increasing ZrO_2_ loading on photon attenuation. The variation in MAC values reflects the changes in the primary photon–matter interaction mechanism across various energy regions. In the low-energy region, attenuation is governed mainly by the photoelectric effect, whose probability exhibits an approximate dependence on *Z^n^* (where *n* typically ranges from 4 to 5) and E^−3.5^ [46]. Since Zr has an atomic number of 40, substantially exceeding those of the elements forming neat VE, its incorporation increases the likelihood of photoelectric absorption in this region. The sharp increases observed in the MAC curves of the ZrO_2_-containing composites at approximately 0.018 MeV are associated with the K-edge of zirconium [47]. The absence of this feature in pure VE further confirms that the observed discontinuity originates from the presence of ZrO_2_. At this energy, the photon energy becomes sufficient to eject K-shell electrons from Zr atoms, leading to a rapid increase in the photoelectric effect probability. Moreover, the magnitude of the K-edge-related enhancement becomes more pronounced with increasing ZrO_2_ loading, consistent with the progressively higher Zr weight fraction in the composites.

At intermediate energies, Compton scattering becomes dominant. Its atomic cross section decreases as photon energy rises and shows an approximately linear dependence on Z [48]. Accordingly, the differences in MAC values among the studied composites gradually diminish with increasing photon energy, suggesting that the influence of atomic number on photon attenuation becomes less pronounced as the prevailing interaction process transitions from photoelectric effect to Compton scattering. At high energy levels, the MAC values of all investigated composites converge, indicating that the influence of ZrO_2_ loading on photon attenuation becomes less significant at these energies. At higher photon energies, photon attenuation is increasingly influenced by pair production, whose interaction probability varies approximately with the square of the atomic number (Z^2^) [29,49]. Overall, the increase in MAC associated with ZrO_2_ incorporation was greatest at low photon energies, whereas the differences among the composites remained comparatively small in the intermediate- and high-energy regions. This energy-dependent behavior indicates that ZrO_2_ incorporation is particularly advantageous for enhancing the attenuation capability of the VE matrix in the low-photon-energy region.

Table 2 presents a comparison of the MAC values obtained from MCNP6.3 simulations, XCOM, and Phy-X/PSD at five selected photon energies (81, 356, 662, 1173, and 1332 keV) associated with ^133^Ba, ^137^Cs, and ^60^Co gamma-ray sources.

The MAC values obtained from MCNP6.3 simulations at selected photon energies showed close agreement with the corresponding XCOM and Phy-X/PSD results. The mean absolute relative deviation was 0.78% for both comparisons, with standard deviations of 0.32% and 0.31% for XCOM and Phy-X/PSD, respectively. The individual deviations ranged from 0.27% to 1.23%. The XCOM and Phy-X/PSD results were also mutually consistent at the selected photon energies, with a mean relative deviation of only 0.02%. These findings support the reliability of the MCNP6.3 model and demonstrate the consistency of the attenuation calculations across the three approaches. ZrO_2_ incorporation markedly increased the MAC at 81 keV, whereas the differences among the composites were small at the higher selected energies. At a photon energy of 81 keV, the MCNP6.3-derived MAC of the VE-Zr20 was approximately 2.04 times that of VE-Zr0. Among the investigated composites, the VE-Zr20 composite showed the maximum MAC values at low photon energies owing to its higher ZrO_2_ content. To place the present results in context, the XCOM-calculated MAC values of VE-Zr20 were compared with literature-reported values for selected polymer-based shielding materials at the same photon energies, as summarized in Table 3. The literature-reported MAC values presented in Table 3 were obtained using different approaches, including experimental measurements, theoretical calculations, and simulations. In contrast, the corresponding MAC values of VE-Zr20 used in this comparison were calculated using XCOM. Therefore, the comparison was performed at the same photon energies, although the experimental and simulation conditions, including the source–sample–detector geometries, were not identical across the studies.

VE-Zr20 exhibited a MAC value approximately 132% higher than that of Benecel K4M/6 wt.% ZnO at 59.5 keV and approximately 43% higher than that of epoxy/30 wt.% Yahyali stone at 81 keV. Its MAC at 81 keV was also nearly identical to that reported for the polyester composite containing 9.13 wt.% PbO, differing by only 0.48%. Thus, a comparable MAC was obtained at this energy without using a lead-based filler, thereby avoiding the associated toxicity and environmental concerns. At 50 and 500 keV, the MAC values of VE-Zr20 were 7.6% and 8.1% lower than those reported for the WO_3_- and Bi_2_O_3_-filled composites, respectively, while its MAC at 662 keV was approximately 2.7 times that reported for the SBR/montmorillonite/MoO composite. Overall, these comparisons indicate that VE-Zr20 provides competitive mass attenuation performance, particularly in the low-energy region, while maintaining a lead-free composition.

**Figure 6:**
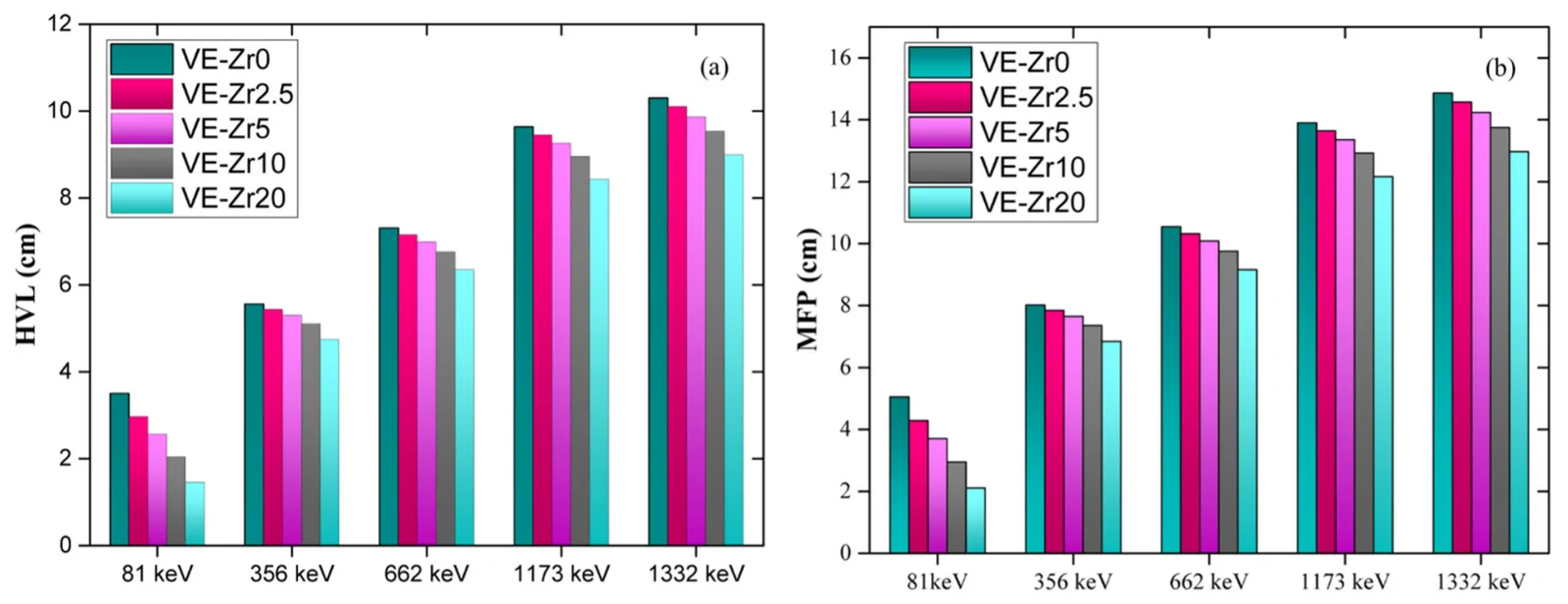
MCNP6.3-derived (**a**) half-value layer and (**b**) mean free path values of the VE–ZrO_2_ composites at the selected photon energies.

To gain further insight into the gamma-ray shielding behavior of the investigated composites, the HVL and MFP parameters were also determined. Figure 6 presents the HVL and MFP values derived from the MCNP6.3 results at the five selected photon energies.

As expected, increasing photon energy resulted in higher HVL and MFP values, reflecting the increased shielding thickness required to achieve the same attenuation level. Conversely, increasing the ZrO_2_ content reduced these parameters. Consequently, the VE-Zr20 composite exhibited the lowest HVL and MFP values at all five selected photon energies, while VE-Zr0 showed the highest values. For example, at 81 keV, the HVL decreased from 3.50 cm for VE-Zr0 to 1.46 cm for VE-Zr20, while the corresponding MFP decreased from 5.05 to 2.11 cm, representing reductions of approximately 58% in both parameters. The pronounced improvement at low photon energies is consistent with the MAC results and can be attributed to the strong Z-dependence of the photoelectric effect. The incorporation of ZrO_2_ increases the contribution of the relatively high-Z Zr atoms to photon interactions, thereby enhancing attenuation and reducing the shielding thickness required. To further assess the shielding efficiency of VE-Zr20, its HVL values were compared with those reported for selected polymer-based shielding composites. At 81 keV, the HVL of the VE-Zr20 composite was 1.46 cm, approximately 56% lower than the value of 3.30 cm reported by El-Khatib et al. for an HDPE composite containing 30 wt.% micro-MgO [56]. Accordingly, the HDPE/MgO composite requires approximately 2.3 times the material thickness of VE-Zr20 to reduce the incident photon intensity by half at this energy. At 662 keV, VE-Zr20 exhibited an HVL of 6.35 cm. At the same energy, Mursaliyev et al. reported HVL values of 6.76, 6.60, and 6.31 cm for PMMA nanocomposites containing 10 wt.% Y_2_O_3_, La_2_O_3_, and Lu_2_O_3_, respectively [57]. The corresponding values for epoxy-based nanocomposites were 6.62, 6.57, and 6.40 cm for 10 wt.% Y_2_O_3_, La_2_O_3_, and Lu_2_O_3_, respectively. Thus, VE-Zr20 exhibited a lower HVL than the Y_2_O_3_- and La_2_O_3_-containing PMMA composites and all three epoxy-based systems, while its HVL was slightly higher than but comparable to that of the Lu_2_O_3_-containing PMMA composite (6.35 vs. 6.31 cm). These results demonstrate the competitive shielding efficiency of VE-Zr20, particularly considering its lead-free composition.

**Figure 7:**
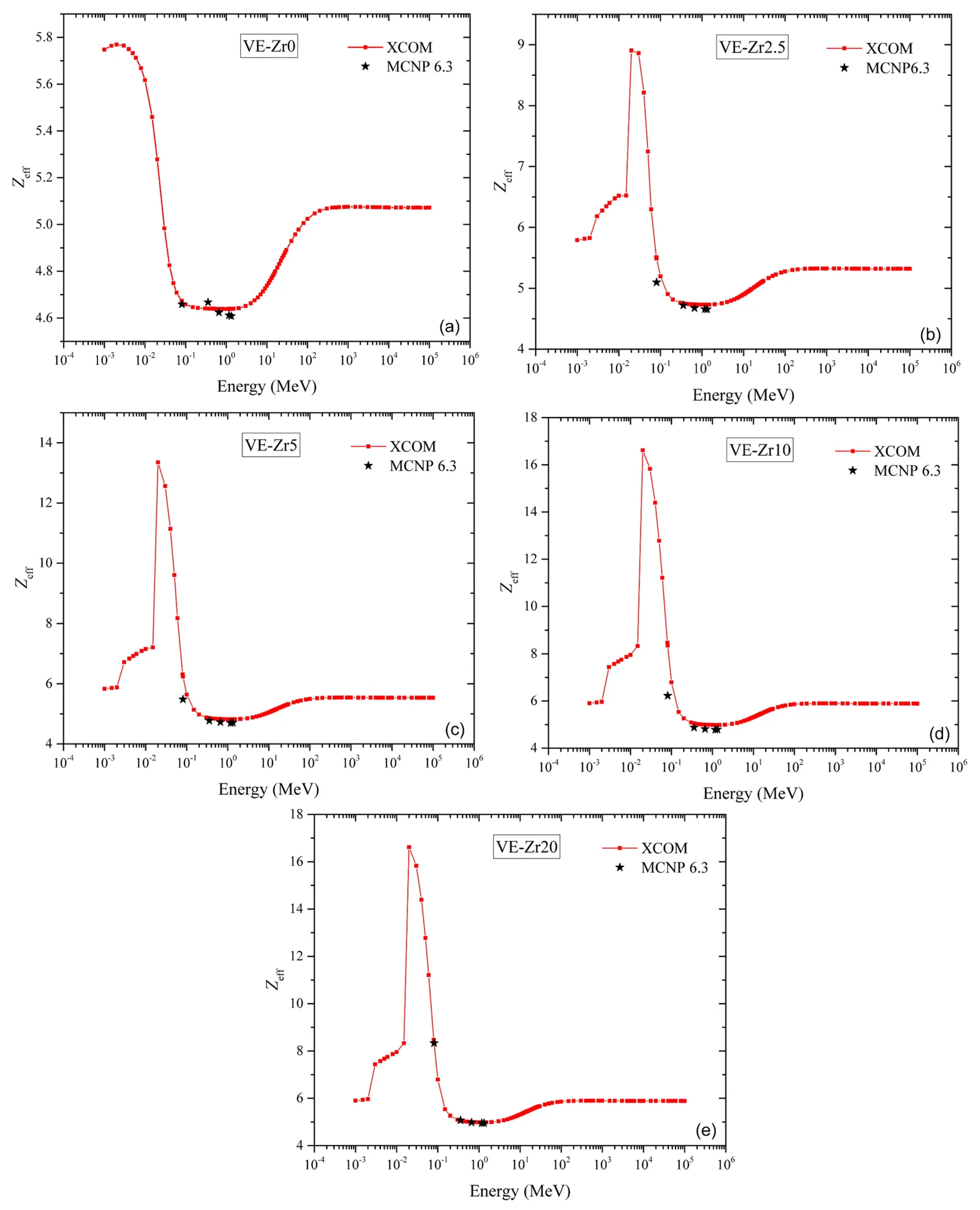
Energy-dependent *Z*_eff_ values of (**a**) VE-Zr0, (**b**) VE-Zr2.5, (**c**) VE-Zr5, (**d**) VE-Zr10, and (**e**) VE-Zr20 derived from XCOM MAC data over 1 keV–100 GeV, together with the corresponding values derived from MCNP6.3 MAC data at five selected photon energies. The sharp changes observed in the low-energy region are associated with the Zr K-edge absorption at approximately 18 keV.

To further elucidate the influence of ZrO_2_ incorporation on the photon attenuation behavior of the composites, the effective atomic number (*Z*_eff_), which reflects the overall photon interaction characteristics of a multielement material, was also evaluated as a function of photon energy. In this context, Figure 7 presents the energy-dependent *Z*_eff_ values derived from XCOM MAC data over 1 keV–100 GeV, together with the corresponding values derived from MCNP6.3 MAC data at the five selected photon energies.

The results showed that *Z*_eff_ exhibited a pronounced dependence on both photon energy and ZrO_2_ content. At a given photon energy, *Z*_eff_ generally increased with increasing ZrO_2_ loading, with the differences being most evident in the low-energy region. The high *Z*_eff_ values observed in this region are attributed to the predominance of photoelectric absorption, whose cross section varies approximately as *Z^n^* (where *n* typically ranges from 4 to 5) and *E*^−3.5^ [46]. For the ZrO_2_-containing composites, a pronounced discontinuity was observed near the Zr K-absorption edge (approximately 18 keV), which became increasingly prominent with increasing ZrO_2_ loading. This feature arises from a sharp rise in the photoelectric interaction probability once the photon energy surpasses the Zr K-edge energy. Above the absorption edge, *Z*_eff_ decreased rapidly with increasing photon energy as the contribution of photoelectric absorption diminished. With further increases in photon energy, the rate of decrease became less pronounced, consistent with the transition from photoelectric absorption to Compton scattering. Within the intermediate-energy range, where Compton scattering predominates, *Z*_eff_ exhibited relatively small variations with photon energy. At high photon energies, the role of pair production became more significant, resulting in a gradual increase in *Z*_eff_ before it approached an approximately constant value at sufficiently high energies. Among the investigated composites, VE-Zr20 exhibited the highest *Z*_eff_ values in the low-energy region, consistent with its greater photon interaction probability in this region. At 81 keV, the *Z*_eff_ value of VE-Zr20 was approximately 1.79 times that of VE-Zr0. Furthermore, the *Z*_eff_ values derived from MCNP6.3 at the five selected photon energies closely followed the corresponding XCOM results, supporting the consistency between the simulation and theoretical calculations.

In addition to the attenuation parameters described above, EABF and EBF were obtained using Phy-X/PSD to assess the contribution of scattered photons to the gamma-ray shielding behavior of the investigated composites. EABF describes the buildup factor associated with the energy absorbed or deposited within a shielding material, and its response function is based on the energy absorbed by the material itself. By contrast, EBF represents the buildup factor associated with radiation exposure, and its response function is based on the energy absorbed in air [58].

**Figure 8:**
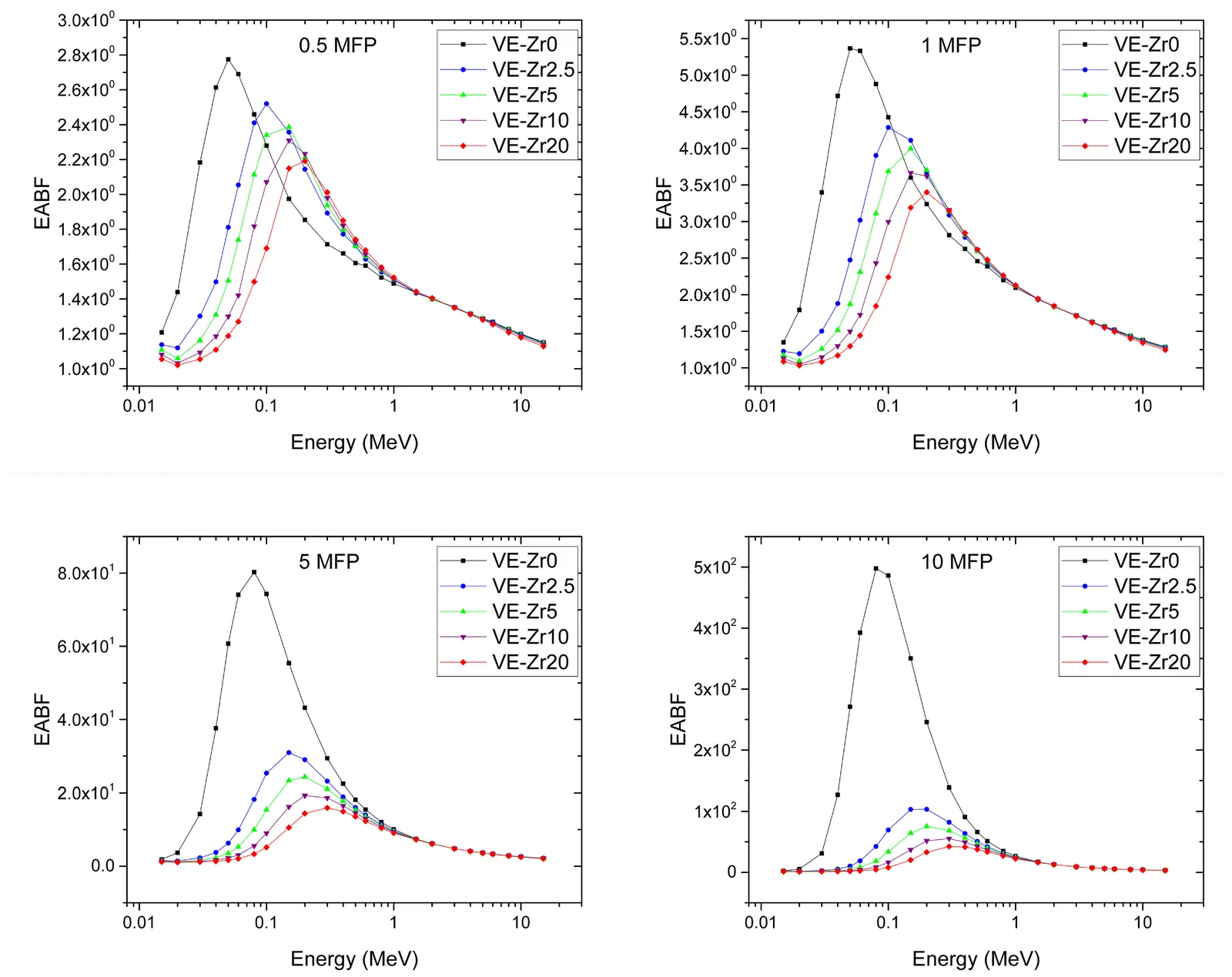

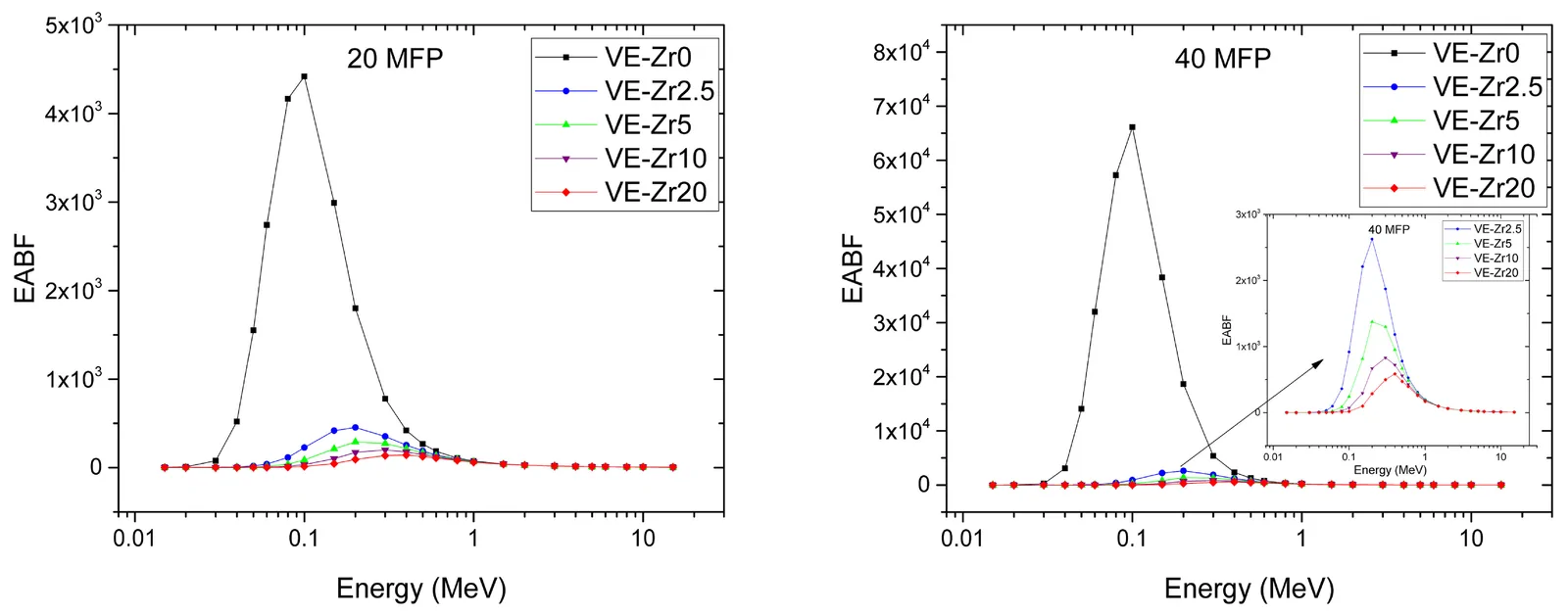
Energy dependence of EABF for VE–ZrO_2_ composites at penetration depths of 0.5, 1, 5, 10, 20, and 40 MFP. The inset in the 40 MFP panel provides an enlarged view of the EABF values for the ZrO_2_-containing composites.

**Figure 9:**
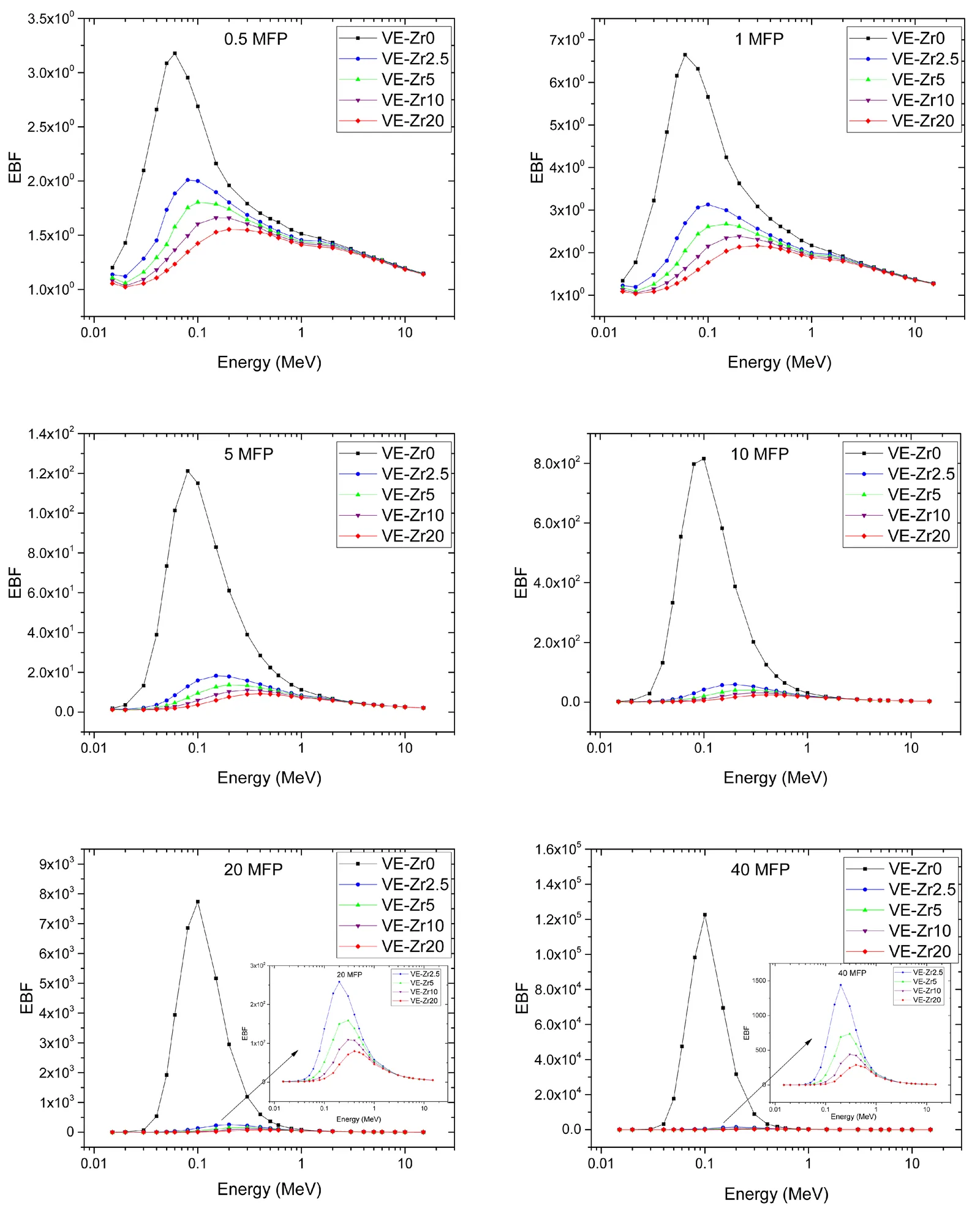
Energy dependence of EBF for VE–ZrO_2_ composites at penetration depths of 0.5, 1, 5, 10, 20, and 40 MFP. The insets in the 20 and 40 MFP panels provide enlarged views of the EBF values for the ZrO_2_-containing composites.

Figure 8 and Figure 9 show the variation in EABF and EBF, respectively, over the photon energy range of 0.015–15 MeV at penetration depths of 0.5, 1, 5, 10, 20, and 40 MFP for the investigated VE–ZrO_2_ composites. Both buildup factors remained low in the low- and high-energy regions but reached maximum values at intermediate photon energies, where Compton scattering was the primary interaction mechanism and the contribution of scattered photons was greatest. At low photon energies, photoelectric absorption predominated, resulting in efficient photon removal and limited buildup. At high photon energies, the increasing contribution of pair production was accompanied by a decrease in both buildup factors. Both EABF and EBF increased markedly with increasing penetration depth because of the greater contribution of multiple scattering along longer photon paths. In addition, the photon energies corresponding to the maximum EABF and EBF values generally shifted toward higher energies with increasing penetration depth. At a given penetration depth, increasing the ZrO_2_ loading reduced the peak EABF and EBF values and shifted the peaks toward higher photon energies. VE-Zr20 exhibited the lowest peak buildup factors among the investigated composites at every penetration depth, although the differences among the composites became small at high photon energies. For example, at 0.5 MFP, the maximum EBF decreased from 3.18 at 0.06 MeV for VE-Zr0 to 2.01 at 0.08 MeV for VE-Zr2.5, 1.80 at 0.10 MeV for VE-Zr5, 1.66 at 0.15 MeV for VE-Zr10, and 1.55 at 0.20 MeV for VE–Zr20. Thus, increasing the ZrO_2_ loading to 20 wt.% reduced the maximum EBF by approximately 51% and shifted its position from 0.06 to 0.20 MeV. A similar reduction in peak magnitude and shift toward higher photon energies were observed for EABF.

## 4. Conclusions

VE-ZrO_2_ composites containing 0–20 wt.% ZrO_2_ were produced, and their gamma radiation shielding behavior was evaluated using MCNP6.3 simulations, with the results benchmarked against XCOM and Phy-X/PSD calculations. At selected photon energies, the MAC values obtained from MCNP6.3 showed close agreement with the corresponding XCOM and Phy-X/PSD results. The mean relative deviation was 0.78% for both comparisons, and the individual deviations ranged from 0.27% to 1.23%. The investigated shielding parameters exhibited a pronounced dependence on both photon energy and ZrO_2_ content. The effect of ZrO_2_ incorporation was most pronounced in the low-energy region, where VE–Zr20 exhibited the highest MAC and the lowest HVL and MFP values among the investigated compositions. At 81 keV, increasing the ZrO_2_ content from 0 to 20 wt.% increased the MCNP6.3-derived MAC by 104.1%, while reducing the HVL from 3.50 to 1.46 cm, corresponding to a decrease of approximately 58%. At the same energy, the Z_eff_ value of VE-Zr20 was approximately 1.79 times that of VE-Zr0, consistent with its enhanced low-energy attenuation in the photoelectric absorption region. The EABF and EBF analyses further demonstrated the influence of ZrO_2_ incorporation on photon buildup behavior. Increasing the ZrO_2_ content reduced the peak buildup factors and shifted their maxima toward higher photon energies. For example, at 0.5 MFP, the maximum EBF decreased from 3.18 for VE-Zr0 to 1.55 for VE-Zr20, corresponding to a reduction of approximately 51%. Taken together, the results demonstrate that ZrO_2_ is an effective filler for improving the low-energy photon attenuation of vinyl ester composites, with VE-Zr20 providing the most favorable low-energy shielding performance among the investigated compositions. These findings support the potential of ZrO_2_-reinforced vinyl ester composites as lightweight, lead-free shielding materials, particularly for applications involving low-energy photons.

## Figures and Tables

**Table 1:** Elemental weight fractions, experimental and theoretical densities, and relative heaviness (RH) values of the investigated pure VE and the VE-ZrO_2_ nanocomposites.

Sample	Elemental Weight Fraction				Density (g cm^−3^)		RH (%)
	H	C	O	Zr	Exp.	Theo.	
VE-Zr0	0.073345	0.71499	0.21166	–	1.152	1.152	10.16
VE-Zr2.5	0.071614	0.69812	0.21280	0.017471	1.179	1.175	10.39
VE-Zr5	0.069956	0.68196	0.21388	0.034203	1.209	1.198	10.66
VE-Zr10	0.066868	0.65186	0.21590	0.065370	1.257	1.242	11.09
VE-Zr20	0.061448	0.59902	0.21945	0.12008	1.349	1.329	11.90

**Table 2:** MAC data from MCNP6.3, XCOM, and Phy-X/PSD for the VE–ZrO_2_ compositions at the selected energies (cm^2^ g^−1^).

Sample	Method	81 keV	356 keV	662 keV	1173 keV	1332 keV
VE-Zr0	MCNP6.3	0.17181	0.10824	0.08231	0.06242	0.05840
	XCOM	0.17270	0.10720	0.08275	0.06305	0.05906
	Phy-X/PSD	0.17269	0.10719	0.08277	0.06305	0.05904
VE-Zr2.5	MCNP6.3	0.19773	0.10819	0.08220	0.06221	0.05822
	XCOM	0.19870	0.10720	0.08255	0.06285	0.05888
	Phy-X/PSD	0.19874	0.10723	0.08257	0.06285	0.05885
VE-Zr5	MCNP6.3	0.22298	0.10811	0.08199	0.06192	0.05810
	XCOM	0.22370	0.10730	0.08236	0.06266	0.05870
	Phy-X/PSD	0.22368	0.10727	0.08237	0.06266	0.05868
VE-Zr10	MCNP6.3	0.26937	0.10805	0.08156	0.06155	0.05784
	XCOM	0.27010	0.10730	0.08200	0.06231	0.05837
	Phy-X/PSD	0.27014	0.10734	0.08201	0.06231	0.05835
VE-Zr20	MCNP6.3	0.35067	0.10824	0.08090	0.06094	0.05713
	XCOM	0.35170	0.10750	0.08137	0.06169	0.05779
	Phy-X/PSD	0.35169	0.10748	0.08139	0.06169	0.05777

**Table 3:** Comparison of XCOM-calculated MAC values for VE–Zr20 with those reported for selected polymer-based shielding materials. The last column presents the MAC values calculated for VE–Zr20 in this study at the corresponding photon energies.

Reference	Photon Energy (keV)	Material Reported in the Literature	Literature MAC (cm^2^ g^−1^)	MAC (cm^2^ g^−1^) for VE-Zr20 (This Study)
[50]	50	Polyester/15 wt.% WO_3_	0.9967	0.9208
[51]	59.5	Benecel K4M/6 wt.% ZnO	0.2700	0.6273
[52]	81	Epoxy/30 wt.% Yahyali stone	0.2460	0.3517
[53]	81	Polyester/9.13 wt.% PbO	0.3534	0.3517
[54]	500	PMMA/20 wt.% Bi_2_O_3_	0.1005	0.0924
[55]	662	SBR/montmorillonite/MoO	0.0304	0.0814

## Data Availability

The original contributions presented in this study are included in the article. Further inquiries can be directed to the corresponding author.

## Abbreviations

The following abbreviations are used in this manuscript:

ZrO_2_	Zirconium oxide
VE	Vinyl ester
TEM	Transmission electron microscopy
XRD	X-ray diffraction
MEKP	Methyl ethyl ketone peroxide
MCNP	Monte Carlo N-Particle
NIST	National Institute of Standards and Technology
XCOM	Photon Cross Sections Database
Phy-X/PSD	Online photon-shielding and dosimetry platform
MAC	Mass attenuation coefficient
LAC	Linear attenuation coefficient
HVL	Half-value layer
MFP	Mean free path
*Z*_eff_	Effective atomic number
EBF	Exposure buildup factor
EABF	Energy absorption buildup factor
